# Research Progress of Ferroptosis in Adiposity-Based Chronic Disease (ABCD)

**DOI:** 10.1155/2022/1052699

**Published:** 2022-04-23

**Authors:** Huijun Du, Xiaoying Ren, Juncai Bai, Wei Yang, Yunan Gao, Shuang Yan

**Affiliations:** ^1^Department of Cardiology, The Fourth Affiliated Hospital of Harbin Medical University, Harbin, 150001 Heilongjiang, China; ^2^Department of Endocrinology, The Fourth Affiliated Hospital of Harbin Medical University, Harbin, 150001 Heilongjiang, China

## Abstract

Ferroptosis is a multistep regulated cell death process induced by iron accumulation and lipid peroxidation. Classical GPX4-dependent pathway and GPX4-independent pathways can independently and synergistically inhibit ferroptosis and jointly maintain the oxidative balance of the body. WHO defines obesity as “a condition of abnormal or excessive fat accumulation in adipose tissue, to the extent that health may be impaired,” and obesity is also defined as an adiposity-based chronic disease (ABCD). Obesity is a systemic disease that leads to metabolic abnormalities in various systems, resulting in a series of complications including obesity cardiomyopathy, atherosclerosis, nonalcoholic fatty liver disease, and diabetes mellitus. Emerging evidence shows that ferroptosis is closely associated with the occurrence and progression of various diseases. In recent years, ferroptosis has been found to play critical roles in obesity and its complications. This review discusses the mechanisms of how ferroptosis is initiated and controlled and discusses the research progress of ferroptosis in obesity and its complications.

## 1. Introduction

Based on functional differences, cell death can be divided into accidental cell death (ACD) and regulatory cell death (RCD): ACD is an uncontrolled process of cell death, triggered by an unexpected injurious stimulus. When the injurious stimulus exceeds the cell's ability to regulate, cell death occurs. RCD involves a tightly structured signal cascade and a molecular-defined effect mechanism, which can be regulated and has unique morphological, immunological, and biochemical characteristics. RCD, which occurs under physiological conditions, is also called programmed cell death (PCD).

For a long time, PCD is regarded as apoptosis. Until recent years, a large number of studies have shown that apoptosis is only one of the death modes of PCD. There is other nonapoptotic form of PCD in cells.

Ferroptosis also meets this criterion for PCD: it is driven by iron-dependent lethal lipid peroxidation (the result of cellular metabolism imbalance and REDOX imbalance), and it can be inhibited either by directly preventing lipid peroxidation or by depleting iron through gene therapy and drug therapy. The main mechanism of ferroptosis is depletion of glutathione and decrease of GPX4 activity, lipid oxides cannot be metabolized through glutathione reductase reaction catalyzed by GPX4, and then iron (II) ion oxidizes lipid to produce reactive oxygen species (ROS), thus promoting cell death. The cellular microstructures showed loss of membrane integrity, cytoplasmic and organelle swelling, and moderate chromatin condensation. The characteristics of mitochondria are reduced mitochondrial volume, less cristae, and membrane density increased.

## 2. System x_c_^−^

The xc cystine/glutamate-exchange transporter is a heterodimer composed of the xCT light chain (slc7a11 gene encodes) and the 4F2 heavy chain (SLC3A2 gene encodes) [1]. The 4F2 heavy chain is a ubiquitously expressed cell surface component shared with several other amino-acid transport systems. The xCT light chain consists of 12 putative transmembrane domains, is selective for cystine and glutamate, and is linked to the 4F2 heavy chain through an extracellular disulfide bond. System x_c_^−^ exchanges intracellular glutamate for extracellular cystine at a ratio of 1 : 1. The endogenous cysteine is largely generated via GSH- or thioredoxin reductase 1- (TXNRD1-) mediated reduction of cystine. GSH is synthesized from glutamic acid, cysteine, and glycine, which are catalyzed by glutamate cysteine ligase (GCL) and GSH synthetase (GSS). The lipid repair enzyme glutathione peroxidase 4 (GPX4) is a central enzyme protecting lipids from oxidative species that uses GSH as an essential cofactor to convert lipid hydroperoxides to lipid alcohols. Interestingly, previous studies have found that system x_c_^−^ drives a cystine/cysteine cycle consisting of cystine import, intracellular reduction of cystine to cysteine, cysteine secretion, and reoxidation to cystine in the extracellular environment and can effectively protect cells from lipid peroxidation [2]. Overall, inhibition of system x_c_^−^ severely impairs antioxidant mechanisms in cells.

**Figure 1:**
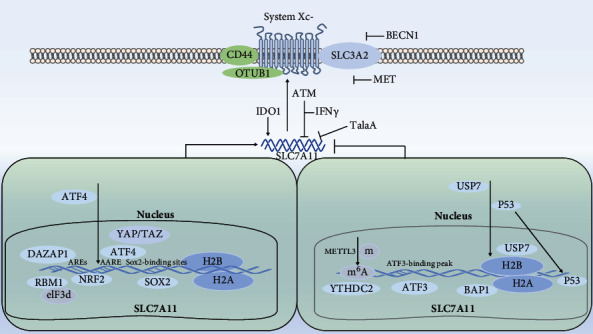
Regulation of SLC7A11: (1) inhibition: ATF3, P53, BAP1, YTHDC2, ATM, IFN*γ*, and MET; (2) upregulation: Nrf2, ATF4, SOX2, RBM1, DAZAP1, and IDO1.

Transcription of the xCT gene and xc transport activities is induced by oxidative stress, mediated by electrophilic agents, depletion of cystine and by oxygen. Further, expression and activity of SLC7A11 are positively regulated by Nrf2, SOX2, RBMS1, and OTUB1, while negatively regulated by P53 [3], ATF3, IFN *γ*, TGF1, ATM, BAP1, and BECN1. This dual regulation constitutes a fine-tuning mechanism to control glutathione levels during ferroptosis (Figure 1).

In response to oxidative stress, the transcription factor Nrf2 binds to antioxidant response elements (AREs) in the Slc7a11 promoter region to promote its transcriptional activities. The basal mRNA level of Slc7a11 may be controlled by activating transcription factor 3 (ATF3) [4]. This is done by ATF3 through binding to the SCL7A11 promoter at BS-1/BS-2 sites of ATF3-binding peak and repressing the expression of SLC7A11. Binding of ATF4 to DNA fragments containing the canonical amino acid responsive element (AARE) binding motif in the promoters of SLC7A11 and ATF3. The transcription factors YAP/TAZ stabilize ATF4 protein and direct it into the nucleus and significantly enrich the AARE motif in the SLC7A11 gene promoter. Together, the results suggest that YAP/TAZ and ATF4 collaboratively regulate SLC7A11 expression [5]. The transcription factor SOX2 can bind a potential specific SOX2-binding site in SLC7A11 promoter and effectively upregulate SLC7A11 expression in a dose-dependent manner [6]. p53 can not only repress transcription of the SLC7A11 gene through a p53-responsive element in the 5′ flanking region [7] but also negatively regulate H2Bub1 to downregulate the expression of SLC7A11 by interacting and promoting the nuclear translocation of USP7 [8]. BRCA1-Associated Protein 1 (BAP1) decreases H2Aub occupancy on the SLC7A11 promoter and represses SLC7A11 expression in a deubiquitinating-dependent manner [9]. The RNA-binding protein RBMS1 can interact with the translation initiation factor eIF3d directly to bridge the 3′- and 5′-UTR of SLC7A11 to promote SLC7A11 transcription [10]. The RBP DAZAP1 (deleted in azoospermia-associated protein 1) interacted with the 3′UTR (untranslated region) of SLC7A11 mRNA and positively regulated its stability. The deubiquitinating enzyme OTUB1 interacts with the N-terminal domain of SLC7A11 to prevent it from degradation [11]. In contrast, the C-terminal domain of SLC7A11 is critical for binding the adhesion molecule CD44. CD44 can modulate its protein stability and enhance the OTUB1-SLC7A11 interaction. The m6A reader protein YTHDC2 promotes the decay of m6A-modified SLC7A11 mRNA by binding to the m6A methylation sites of the 3′UTR of SLC7A11 mRNA [12]. YTHDC2 m6A-dependently destabilized homeobox A13 (HOXA13) mRNA because a potential m6A recognition site was identified within its 3′ untranslated region (3′UTR). Interestingly, HOXA13 acted as a transcription factor to stimulate SLC3A2 expression. Thereby, YTHDC2 suppressed SLC3A2 via inhibiting HOXA13 in an m6A-indirect manner [13].

Indoleamine-2,3-dioxygenase 1 deficiency activate SLC7A11 expression. Talaroconvolutin A (TalaA), a novel ferroptosis inducer, can downregulate of SLC7A11. The autophagy-related protein Beclin1 (BECN1) [14] and SLC7A11 trigger formation of the BECN1-SLC7A11 complex through protein-protein interactions to inhibit system x_c_^−^ activity but not the expression level of SLC7A11. Radiation-activated ATM and IFN*γ*-mediated JAK-STAT1 signaling pathways independently and cooperatively inhibit SLC7A11 [15]. Metformin reduces the protein stability of SLC7A11 by inhibiting its UFMylation process [16] (Figure 1).

mir-182-5p and mir-378A-3p negatively regulate the expression of GPX4 and SLC7A11 by directly binding to the 3′UTR of GPX4 and SLC7A11 mRNA [17]. Researches showed that miR-375 [18], miR-27a [19], miR-26b [20], and As-SLC7A11 (antisense lncRNAs) [21] could suppress the transcription of SLC7A11 mRNA. In addition, knockdown of FTH1 leads to downregulation of Slc7a11 mRNA [22].

## 3. Ferroptosis Defense Systems

GPX4 is the major enzyme catalyzing the reduction of PLOOHs in mammalian cells, which can reduce the toxicity of the peroxides and maintain the homeostasis of lipid bilayer membranes through its enzymatic activity.

Selenium can activate the transcription Sp1 to upregulate GPX4 in neurons. GSH is a cofactor of GPX4. GPX4 is irreversibly inactivated when GSH is depleted, because it uses GSH as a reducing agent during the peroxidase reaction cycle. G-rich RNA sequence binding factor 1 (GRSF1) binds to the 5′UTR of the mitochondrial GPX4 mRNA and upregulates its translation. Inhibition of mevalonate pathway can downregulate the selenocysteine tRNA (tRNA (Sec)) to affect GPX4 protein synthesis. DMOCPTL induced GPX4 ubiquitination by directly binding to GPX4 protein, while HSPA5, also known as GRP78 or BIP, can interact with GPX4 to limit GPX4 ubiquitination degradation. Moreover, RSL3 binding to GPX4 completely inhibits the activity of GPX4, and doxorubicin can downregulate GPX4 expression. QD394 and QD394-Me (namely, QD430) representing novel ROS-inducing drug-like compounds were confirmed to inhibit GPX4 activity. mir-182-5p and mir-378A-3p negatively regulate the expression of GPX4 and SLC7A11 by directly binding to the 3′UTR of GPX4 and SLC7A11 mRNA. Circular RNA circKIF4A suppresses ferroptosis by sponging miR-1231 and upregulating GPX4.

There were at least five defense systems, in addition to GPX4–GSH axis, there were FSP1-COQ10-NAD(P)H pathway, DHODH-mediated ferroptosis defense, GCH1–BH4–DHFR axis, and ESCRT III-mediated plasma membrane repair system.

The CoQ oxidoreductase FSP1 acts parallel to GPX4 to inhibit ferroptosis. Ferroptosis is suppressed when a myristoylated version of FSP1 is recruited to the plasma membrane where it reduces CoQ10 to CoQ10-H2 that traps lipid peroxides. In addition, FSP1 was found to be closely related to endoplasmic reticulum (ER), Golgi complex, and organelle membrane [23, 24]. DHODH indirectly promotes the regeneration of CoQ-H2 by reducing FMN and inhibits lipid peroxidation in mitochondria, a pathway independent of mitochondrial GPX4. The increase in DHODH activity increased the resistance to ferroptosis in cell lines overexpressing GPX4 and FSP1 [25]. The GCH1-BH4-phospholipid axis acts as a master regulator of ferroptosis resistance, controlling endogenous production of the antioxidant BH4, abundance of CoQ10, and peroxidation of unusual phospholipids with two polyunsaturated fatty acyl tails [26]. It was found that an increase in the concentration of free intracytoplasmic Ca2+, cell swelling, and plasma membrane rupture were important markers in ferroptosis, and that before plasma membrane rupture, and that before plasma membrane rupture, cytoplasmic free Ca2+ concentration and lipid oxidation increased and confirmed the pore size of the plasma membrane can be estimated to be approximately 2.5 nm. Further studies showed that Ca 2+ influx-mediated activation and recruitment of ESCRT-III complex leads to the repair of damaged plasma membranes during cell death and also regulates cytokine secretion in ferroptotic cells.

**Figure 2:**
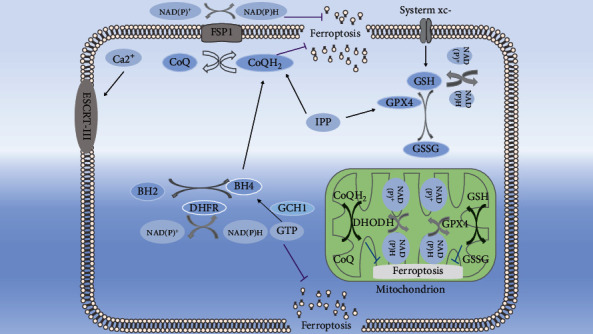
Defense systems: (1) GPX4–GSH axis; (2) FSP1-COQ10-NAD(P)H pathway; (3) DHODH-mediated ferroptosis defense; (4) GCH1–BH4–DHFR axis; (5) ESCRT III-mediated plasma membrane repair system.

FSP1 can activate the ESCRT III-mediated plasma membrane repair. GPX4 and FSP1 seem to simultaneously overlap with the mevalonate. The mevalonate pathway is crucial for the synthesis of GPX4 itself and generation of the CoQ10 backbone. IPP produced by the mevalonate pathway is the precursor of CoQ10. IPP positively regulates Sec-tRNA, which functions as a key regulatory element during the maturation of GPX4pathway, while CoQ10 is the main effector of the FSP1 pathway (Figure 2). More recently, the small molecule FIN56, a potent inducer of ferroptosis, appeared to induce ferroptosis through a dual mechanism of depleting GPX4 protein and coenzyme Q10 [27]. In contrast to previously described ferroptosis inducers, FINO2 does not inhibit system x_c_^−^ or directly target GPX4, nor does it induce ferroptosis through ALOX [28].

## 4. Lipid Metabolism

Lipid peroxidation is a hallmark of ferroptosis and directly destroys cellular membranes, thereby causing ferroptosis. Inhibition of GSH synthesis and of acquisition of cystine from the extracellular environment, or direct inhibition of GPX4 activity, results in the accumulation of lipid peroxides of cellular membranes, thereby leading to ferroptosis.

Lipid peroxidation is achieved by two main pathways, by enzymatic or by nonenzymatic oxidation, respectively. Enzymatic lipid peroxidation is mediated in a controlled manner by the activity of the lipoxygenase (LOX) family, which can oxidize free and esterified PUFA to produce peroxide radicals. Acyl-CoA Synthetase Long Chain Family Member 4 (ACSL4) and lysophosphatidylcholine acyltransferase 3 (LPCAT3) promote polyunsaturated fatty acids (PUFAs) to incorporate into phospholipids form polyunsaturated fatty acid phospholipids (PUFA-PL). PUFA-PL is prone to Arachidonic acid lipoxygenases (ALOXs) induced lipid peroxidation, which ultimately leads to the destruction of lipid bilayers and affects cell membrane function, thereby promoting ferroptosis.

ACSL4 expression can be upregulated by radiation therapy, and ACSL1 is responsible for ferroptosis induced by conjugated linoleates including *α*-eleostearic acid (*α*ESA). In contrast, ACSL3 converts monounsaturated fatty acids (MUFAs) into their acyl-CoA esters for incorporation into membrane phospholipids, thus protecting cancer cells against ferroptosis. In addition, vitamin E can inhibit the ALOX activity, and CAFs secrete exosomal miR-522 to inhibit ferroptosis in cancer cells by targeting ALOX15 and blocking lipid-ROS accumulation.

Interestingly, the LOX activator alone did not cause ferroptosis, but overexpression of ALOX increased drug-induced cell death [29]. ALOX was also confirmed to be required for ferroptosis under GSH depletion conditions [30]. Thus, LOXs may not be the key drivers of ferroptosis in most contexts but could contribute to the initiation and/or propagation of the damage in some contexts. LOXs combine with PEBP1 to form the 15-LOX/PEBP1 complex; simultaneously, allosteric regulation forms the ferroptosis marker signal 15-HpETE-PE, thus initiating ferroptosis. Calcium-independent phospholipase A2*β* (iPLA2*β*) preferentially hydrolyzes peroxidized PLs, such as 15-HpETE-PE, and Fer-1 can directly bind to and interfere with the 15-LOX/PEBP1 complex, effectively inhibit HpETE-PE and inhibit ferroptosis. In addition, the ALOX15 protein consistently localizes to cell membrane during the course of ferroptosis, and its localization and its interaction with lipid membranes are critical for ferroptosis.

Nonenzymatic lipid peroxidation or spontaneous lipid peroxidation is a free radical driven chain reaction in which one free radical induces the oxidation of lipids. The iron ions entering the cells can react with reactive oxygen species, resulting in the peroxidation of polyunsaturated fatty acids and the formation of phospholipid hydroperoxides (PLOOHs), when GPX4 is inhibited, PLOOHs can persist longer, initiating the Fenton reaction to rapidly amplify PLOOHs. PLOOHs can react with both ferrous and ferric ions to generate the free radicals PLO • and PLOO •, respectively, driving the damaging peroxidation chain reaction that disrupts the structure of cell membranes and ultimately leads to ferroptosis.

## 5. Iron Metabolism

A certain amount of free iron is fundamental to the occurrence of ferroptosis, and iron chelating agents (such as DFO) can reduce nearly all of lipid peroxides. In addition, iron can increase the activity of arachidonate lipoxygenase (ALOX). Due to the central role of iron in ferroptosis, ferroptosis sensitivity can be altered by changing intracellular free iron.

**Figure 3:**
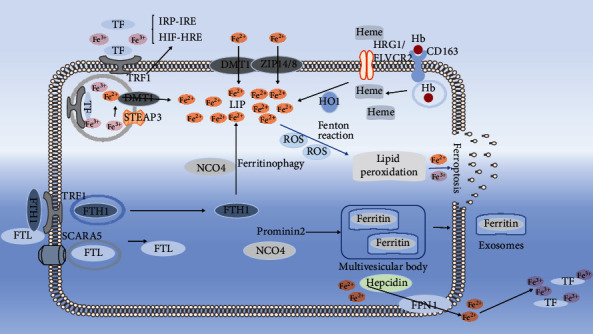
There are several sources of intracellular iron. FPN is the only known iron transporter responsible for cellular iron export.

There are several sources of intracellular iron (Figure 3). Ferric iron delivered via transferrin binds to TFR1, then through endocytosis, it is released into the vesicles as Fe3+ and reduced to Fe^2+^ by the ferric reductase six-transmembrane epithelial antigen of the prostate 3 (STEAP3) and is then transported into the cytosol by divalent metal-ion transporter-1 (DMT1). This is the main way of iron absorption, so TRF1 is considered as a ferroptosis marker. Nontransferrin bound iron is reduced by ferric reductase to ferrous iron (Fe^2+^) and can be directly transported into cells by the metal transporters DMT1, ZIP8, and ZIP14. Extracellular hemoglobin is recognized by the scavenger receptor CD163 and endocytosed into cells and further degraded into heme. In addition, circulating free heme can be directly exported to the cytoplasm by HRG1 and FLVCR2, and the heme in the cytoplasm releases Fe^2+^ because of the activity of HO-1. The light and heavy ferritin chains are endocytosed by SCARA5 and TFR1 on the cell membrane, respectively. The iron obtained through DMT1 constitutes the cytoplasmic labile iron pool (LIP) in which iron is metabolically active.

The major iron metabolic proteins, such as TFR1, DMT1, and HPN1, are posttranscriptionally regulated by Iron regulatory protein- (IRP-) iron-responsive element (IRE) and hypoxia-inducible factor- (HIF-) hypoxia-responsive element (HRE) systems. Iron regulatory proteins, including IRP1 and IRP2, play a synergistic role in ferroptosis. IRP1 significantly promoted erastin- and RSL3-induced ferroptosis. IRP2 had a weak effect but could enhance the promoting function of IRP1 on ferroptosis. Further, erastin and RSL3 promoted the transition of aconitase 1 to IRP1. HIF-HRE plays a dual role in ferroptosis, and the mechanism of double action is not limited to regulating the free iron content. The activation of HIF-1*α* and HIF-2*α* in triple negative breast cancer (TNBC) cells correlated with SLC7A11 overexpression. HIF-1*α* can limit ferroptosis by increasing expression of fatty acid-binding protein (FABP3), FABP7, and under hypoxia can decrease ferritin and inhibit autophagic flux under hypoxia. In addition, HIF-1*α* can reduce ferroptosis by inhibiting ACSL4 expression in the early stage of ischemic stroke. HIF-2*α* induces ROS accumulation followed by ferroptosis in colon cancer cells through nonmitochondrial metabolism. HIF-2*α* also increases hypoxia-inducible lipid droplet-associated protein (HILPDA) expression, which promotes enrichment of polyunsaturated lipids and lipid peroxidation and the subsequent ferroptosis. In addition, clear-cell carcinoma cells are intrinsically sensitive to GPX4 inhibition-induced ferroptosis, and this regulation is mediated by HIF-2*α*. Interestingly, there can be mutual inhibition between two systems, that is to say, HIF2*α* translation is inhibited by IRP1, while IRP1 transcription is inhibited by HIF.

The export of iron via ferroportin (FPN, encoded by Slc40a1) is coupled to the reoxidation of Fe^2+^ to Fe^3+^, then bound to transferrin and returned to the circulation (Figure 3). The expression of FPN is regulated by Hepcidin, which is a 25 amino acid peptide coded for by HAMP. In addition, the pentaspan membrane glycoprotein Prominin-2 (Prom2) can export iron from the cell by promoting the formation of ferritin containing multivesicular bodies and exosomes. miR-20a and miR-485-3p can reduce iron output by targeting FPN genes. Ferritinophagy is mediated by nuclear receptor coactivator 4 (NCOA4), which directly binds FTH and transfers the complex to the autolysosome for degradation, which will promote the release of Fe3+ free resulting in the increase of intracellular free iron. Heat shock protein B1 (HSPB1), miR-210, miR-152, and miR-30b-5p inhibit the expression level of TFR, thereby reducing the intracellular iron concentration. Concurrently, the expressions of miR-200b and miR-Let-7d effectively reduce iron accumulation by inhibiting the expression of FTH and DMT1-iron-responsive element (IRE), respectively. The upregulation of ubiquitin-specific processing protease 7 (USP7) accelerates ferroptosis by activating the p53/TfR1 pathway. Cuprizone (CZ) is a copper chelator t, chelating copper with CZ rapidly mobilizes iron from ferritin.

## 6. Copper Metabolism

First, there is metabolic crosstalk between copper and iron, due to that some studies have found that DMT1, DCYTB (Duodenal Cytochrome b), FPN1, HP (Hephaestin), Hepcidin, STEAP proteins, CP(Ceruloplasmin), and ATP7A (ATPase Copper Transporting Alpha) can all participate in iron and copper metabolism [31], and thus copper can regulate ferroptosis through copper-iron interactions. Second, copper is linked to ferroptosis by regulating ROS accumulation in cells, and this mechanism may be independent of iron metabolism itself.

It was found that CuSO4 could induce ferroptosis through autophagy-dependent degradation of ferritin [32]. Combinational treatment of elesclomol and copper could lead to copper retention within mitochondria due to ATP7A downregulation through a protein degradation pathway, leading to ROS accumulation, simultaneously ATP7A loss inhibited the interaction between SLC77A11 and CD44, which in turn promoted the degradation of SLC7A11, thus further enhancing oxidative stress and consequent ferroptosis [33]. Cuprizone (CZ), another copper chelator, could increase the expression of NCOA4, leading to ferritin phagocytosis to release of iron, and iron overload resulting from enhanced iron uptake by high expression of TfR1, thereby induced ferroptosis [34]. COMMD10-N terminus could combine with HIF1*α*, and its low expression could inhibit the ubiquitin degradation of HIF1a (by inducing Cu accumulation), promoting HIF1a nuclear translocation and the transcription of ceruloplasmin (CP) and SLC7A11, which jointly promoted intracellular GSH synthesis and decreased lipid peroxidation, thereby inhibited ferroptosis. In addition, high intracellular copper concentrations elevated CP expression to reduce intracellular concentrations of Fe, decreasing ferroptosis by directly reducing high levels of lipid peroxidation. Upregulation of CP could also enhance HIF1a expression, forming a HIF1a/CP positive feedback regulatory loop [35]. DSF/Cu could intensively impair mitochondrial homeostasis, increase free iron pool, also induce p53 protein expression leading to SAT1 and ALOX15 upregulation, enhance lipid peroxidation, and eventually result in ferroptosis. However, DSF/Cu also compensatively activated p62-Keap1-Nrf2 antioxidant pathway, which in turn showed a protective effect in ferroptosis [36, 37]. In addition, various copper complexes [38] and copper complex nanoformulations [39, 40] have been demonstrated to induce ferroptosis.

## 7. Ferroptosis Spread

Interestingly, cell death induced by “class 2” ferroptosis-inducing compounds (FINs) (directly binding and loss of activity of GPx4) appears to be random cell death, whereas cell death induced by “class 1” FINs (pharmacological inhibiting system x_c_^−^) transmitting from ferroptotic cells to neighboring cells [41]. Further studies showed that propagation of a ferroptosis-inducing signal occurs upstream of cell rupture and involves the spreading of a cell swelling effect through cell populations in a lipid peroxide- and iron-dependent manner. However, a recent study has found that diffusion of ferroptosis is a common phenomenon, with lipid peroxidation and its sequelae spreading from ferroptotic cells to surrounding cells even if peripheral cells are not exposed to FINS. In addition, it was found that 3D spherical cells were subjected to more oxidative stress than a monolayer of cells, the cells inside and outside the sphere had different antioxidant ability, and the outer cells were more able to adapt to increased oxidative stress, while ferroptosis was the way of cell death inside the sphere [42].

Ferroptosis was also negatively correlated with cell density, and the sensitivity of ferroptosis depended on the activity of the Hippo pathway.

Recently, ferroptosis has also been found being regulated by the cellular contact and density through the Hippo-YAP/TAZ-EMP1-NOX4 pathway. The YAP/TAZ activation under low density renders cell sensitivity to ferroptosis, while the YAP/TAZ activity decreased and ferroptosis sensitivity decreased under high density [43]. It was further confirmed that the direct targets of the YPA–TEAD complex were TFRC and ACSL4, and their expression decreased was attributed to an increase in cell density. E-cadherin-mediated epithelial cell-cell contact inhibits ferroptosis by activating the Hippo signaling pathway via ubiquitination of Merlin [43]. Cell density-dependent ferroptosis has also been observed in nonepithelial cells which do not express E-cadherin, suggesting that other cell adhesion molecules may also inhibit ferroptosis through similar mechanism as described above. *α*6 and *β*4 integrin subunits, for example, also prevent ferroptosis in breast cancer cells in vitro.

The epithelial-to-mesenchymal transition (EMT) may also contribute to ferroptosis. The oncogene lysine-rich CEACAM1 coisolated protein (LYRIC, also known as metadherin or astrocyte elevated gene-1) is a positive regulator of EMT and promotes ferroptosis by inhibiting the expression of GPX4 and SLC3A2. Mesenchymal state cells are more prone to ferroptosis due to the loss of cell-cell contacts and the activation of factors involved in EMT programs, such as ZEB1, SNAI1, and TWIST1.

Ferroptosis is ultimately caused by plasma membrane rupture. Redox enzymes cytochrome P450 reductase (POR) and cytochrome B5 reductase 1 (CYB5R1) localized in the endoplasmic reticulum were found to play a major role. Typically, these enzymes normally transfer two electrons from NAD(P)H to downstream proteins such as CYP450. But in the abnormal electron-transfer process, the electrons may be accidentally transferred to oxygen. This converts oxygen into dangerous compounds such as superoxide radicals and hydrogen peroxide, which can react with iron to form hydroxyl radicals. Hydroxyl radicals can remove a hydrogen atom of the double bonds of unsaturated fatty acids, causing lipid peroxidation, and subsequently, promoting cell death.

## 8. An Adiposity-Based Chronic Disease (ABCD)

Obesity is an endocrine, nutritional, and metabolic disease characterized by the excessive accumulation and storage of fat in the body, which can lead to various metabolic abnormalities. The American Association of Clinical Endocrinologists (AACE) and The European Asylum Support Office (EASO) recommend using a new diagnostic term for obesity: adiposity-based chronic disease (ABCD) [44, 45]. The ABCD of obesity has a brand-new disease classification system, including A, B, C, and D, group A code represents the cause of obesity, group B code represents BMI, group C code represents obesity-related complications, and group D code defines the severity of complications. A primary aim of the ABCD is to emphasize a complications-centric approach that primarily determines therapeutic decisions. Complications of obesity include obesity cardiomyopathy, atherosclerosis, nonalcoholic fatty liver disease, and diabetes mellitus. Most of the time, it is these complications, rather than obesity itself, that seriously affect longevity and quality of life of patients. This article reviews the role of ferroptosis in the pathogenesis of obesity and its metabolic complications.

### 8.1. Obesity Cardiomyopathy

Heat shock factor 1 (HSF1) plays a role in the resistance in palmitic acid- (PA-) induced cell death in cardiomyocytes by regulating iron homeostasis and promoting GPX4 expression [46]. PA was found to decrease the protein expression levels of both HSF1 and GPX4 in a dose- and time-dependent manner, which were restored by different ferroptosis inhibitors. GPX4 overexpression protected against PA-induced ferroptosis. Overexpression of HSF1 not only restored GPX4 protein expression but also improved iron overload by regulating the transcription of iron metabolism-related genes (such as FTH1, TFRC, and Slc40a1). In addition, inhibition of endoplasmic reticulum (ER) stress contributed to HSF1-mediated GPX4 expression.

### 8.2. Atherosclerosis

In human and animal models, iron content in atherosclerotic lesions is significantly higher than that in arterial tissue in health. The accumulation of iron in macrophages in atherosclerotic plaques was the most significant and was closely related to the severity of atherosclerosis (AS). Endothelial dysfunction plays a key role in the occurrence and development of AS, and iron overload induces endothelial dysfunction by enhancing the oxidative and inflammatory responses of endothelial cells.

Inhibition of ferroptosis could alleviate AS by reducing lipid peroxidation and endothelial dysfunction. It was found that Ferrostatin-1 (fer-1) and iron-chelating agents could significantly inhibit dyslipidemia and iron overload, the decreased expression levels of SLC7A11, GPX4, and GSH in thoracic aorta of HFD-fed mice [47]. In addition, it was found that HMOX1 was increased by HGHL, which induced iron overload and oxidative stress. More importantly, utilizing HMOX1-specific siRNA (siRNA HMOX1) could inhibit iron levels and enhance the expressions of SLC7A11 and GPX4. Therefore, inhibition of HMOX1 can improve iron death in endothelial cells [47].

Fluvastatin inhibits the ferroptosis of vascular endothelial cells by upregulating GPX4 and xCT [48]. ox-LDL promotes cytokine production and reduces cell proliferation, migration, invasion, and angiogenesis. It also inhibits GPx4 and xCT expression and induces ferroptosis, which can be reversed by deferoxamine mesylate and fluvastatin. Knockdown of GPx4 and xCT expression partially blocked the protective effect of fluvastatin.

SIRT1-autophagy axis inhibits excess iron-induced ferroptosis of foam cells and subsequently increases IL-1*Β* and IL-18 [49]. It was found that excessive iron-induced massive foam cell death and induced expression of proinflammatory cytokines such as IL-1B and IL-1*β*, which could be reversed by SIRT1 inhibition and ferroptosis inhibitors. Further studies showed that SIRT1 reduces foam cell ferroptosis by restoring autophagy flux.

### 8.3. Nonalcoholic Fatty Liver Disease (NAFLD)

The Sirtuin family of proteins plays an increasingly important role in the occurrence and development of NAFLD. Some studies show that activating SIRT1 can reduce iron accumulation in splenic macrophages by inhibiting hepcidin [50] and even inhibit high iron-induced ferroptosis by autophagy [49]. Interestingly, intestinal SIRT1 deficiency may attenuate hepatic CISD1 and/or CISD2 proteins in alcohol-induced liver injury, which in turn blocks the ability of ethanol to release [2Fe-2S] clusters, induce iron accumulation, and generate ROS, and ultimately alleviate ferroptosis. This study suggests that LCN2-SAA1 axis may serve as a pivotal endocrine mediator in the cross-communications between gut and liver in mice following ethanol intake [51]. Both SIRT2 and SIRT3 can inhibit ferroptosis through downregulating p53 [52, 53]. Moreover, SIRT3 can also inhibit AKT-dependent mitochondrial metabolism and promoting AMPK-mTOR pathway and decreasing GPX4 level to induce ferroptosis [54, 55]. SIRT6 inhibition can lead to the inactivation of the Keap1/Nrf2 signaling pathway and downregulation of GPX4, which in turn triggers ferroptosis [56].

Ferroptosis is the initiating cell death in the onset of nonalcoholic steatohepatitis [57, 58], the process facilitated by arachidonic acid metabolism. Ferroptosis inhibitors can reduce hepatic inflammation, liver damage, and fibrosis [59]. In MCD diet model of nonalcoholic steatohepatitis (NASH), two ferroptosis inhibitors, trolox and deferiprone (DFP), can almost completely inhibit inflammatory cell infiltration, cytokine expression, and cell death. However, deferoxamine (DFO) did not improve elevated serum levels of markers of liver injury, and it is likely that membrane permeability of DFP was significantly higher than that of DFO. This means that intracellular iron plays a key role in necrotic cell death. Another study demonstrated increased liver and serum iron levels and enhanced expression of TRF1, FTL, FTH, and FPN in the model. In addition, RNA-seq analysis suggested that elevated arachidonic acid metabolism promotes ferroptosis in MCD-diet fed mouse livers. Lipid ROS promotes liver steatosis by inducing lipid droplet formation, and ferroptosis inhibition significantly reduced hepatic lipid droplets, thereby alleviating hepatic steatosis.

Enoyl coenzyme A hydratase 1(ECH1) may ameliorate NASH by inhibiting ferroptosis [60]. ECH1 was significantly upregulated in liver tissues of patients with NASH and in dietary-induced NASH animal models. In addition, ECH1 overexpression obviously alleviated hepatic lipid accumulation of mice, significantly reduced levels of inflammatory markers, and increased the protein and mRNA levels of GPX4. Knockdown of ECH1 exacerbated the progression of NASH, whereas this effect can be reversed by ferroptosis inhibition.

Ginkgolide B (GB) has an inhibited effect on ferroptosis in NAFLD, possibly through Nrf2 signaling pathway [61]. In an HFD-induced NAFLD mouse model, it was found that GB could significantly reduce iron overload, lipid peroxidation, and the decline in Nrf2 signaling. Further research found that GB could effectively upregulate the expression of GPX4/HO-1 through Nrf2 pathway, improving the tolerance of ferroptosis, and thus reduce lipid accumulation and lessen liver injury.

### 8.4. Diabetes Mellitus

Circulating iron and ferritin levels were significantly elevated in T2DM individuals. Furthermore, a recent study showed that patients with diabetes exhibit significantly lower levels of GPX4 enzyme in their heart than age-matched nondiabetic patients [62]. The tolerance of diabetic myocardium to ischemia-reperfusion injury (IRI) injury is much lower than normal myocardium. Hyperglycemia caused by diabetes can induce oxidative stress by producing ROS through advanced glycation end products, polyol pathway, and de novo synthesis of triose metabolism, which could contribute to the exacerbation of myocardial IRI, when combined with reperfusion injury. Ferroptosis inhibition reduced endoplasmic reticulum stress (ERS) and then alleviated myocardial IRI. The ferroptosis inhibitor Fer-1 alleviated myocardial damage in H9c2 cells in a high glucose condition and prevented H9c2 cell damage during hypoxia/reoxygenation [63].

Ferroptosis plays a key role in the development of diabetic nephropathy. The expression of xCT and GPX4 in the kidney of diabetic mice and in vitro cultured renal tubular epithelial cells was significantly decreased, the level of GSH was low, and lipid peroxidation was enhanced. In addition, iron overload and the characteristic morphological changes of mitochondria in ferroptosis were observed. All these changes induced by can be recovered by Fer-1 treatment. Decreased Nrf2 was also found in the models of diabetic nephropathy. The sensitivity of Nrf2-knockdown cells to ferroptosis is increased, upregulating Nrf2 by treating with fenofibrate could regulate the expression of GPX4, SLC7A11, FTH-1, and TFR-1, which rescued disordered iron pool, thereby inhibiting the diabetes-related ferroptosis and delaying the onset and development of DN. In addition, some studies have shown increased ROS and MDA production and decreased GSH activity, as well as iron overload and downregulated SLC7A11 and GPX4 expression in cultured mice podocytes stimulated with high glucose. In short, renal corpuscle and renal tubule will be affected by high glucose resulting in ferroptosis and kidney damage in diabetic patients [64].

Quercetin ameliorated decreased insulin secretion and insulin resistance by alleviating the ferroptosis in pancreatic *β*-cells in T2DM mice [65]. Despite compensatory increase of xCT, decreased levels of GSH and GPX4 and downregulated levels of mitochondrial outer-membrane protein VDAC2 were still observed. Interestingly, the treatment with quercetin reversed these changes. In addition, iron and ferritin deposition were found in or near the center of islets in diabetic mice. And quercetin could reduce iron level and ferritin deposition. Quercetin also reduced the increased PTGS2 mRNA expression induced by HG in vitro pancreatic cell.

Changes in xCT expression and system x_c_^−^ activity may occur on the early stage of type 2 diabetes. Sustained hyperglycemia can lead to a series of vascular changes, and it is worth noting that neurological changes, such as a reduction in the oscillatory potentials of the electroretinogram, have been shown in patients with diabetes and animal models of diabetes prior to vascular changes. Retinal cells exposed to high glucose also showed decreased system x_c_^−^ activity, lower levels of GSH, and increased oxidative stress early. Human psychophysics indicates that color sensitivity is impaired and could be due to increased oxidative stress in photoreceptor cells.

## 9. Conclusion

Taken together, ferroptosis is the result of the imbalance between oxidation and antioxidant system in the body, and it is mediated by lipid peroxidation and iron overload. Ferroptosis has been reported to be involved in the development of almost all cancers, as well as in the occurrence of cardiovascular disorders, digestive disorders, and diseases of other systems. Therefore, it is considered that ferroptosis is closely related to the endocrinology and metabolism. Previous studies have also confirmed that ferroptosis is involved in glucose and lipid metabolism of the body, and ferroptosis has also been found in various complications of obesity. Inhibition of ferroptosis can significantly improve the pathological damage of damaged tissues. However, the experimental research involving the specific mechanism of ferroptosis in obesity remains lacking, and the specific regulatory molecules need to be further discussed. More comprehensive and in-depth research on ferroptosis in the field of endocrinology and metabolism is needed to consider the benefits of inhibiting ferroptosis to protect damage caused by obesity and to gain benefit in clinical outcomes in the future.

## References

[1] Sato H., Tamba M., Ishii T., Bannai S. (1999). Cloning and expression of a plasma membrane cystine/glutamate exchange transporter composed of two distinct proteins. *The Journal of Biological Chemistry*.

[2] Banjac A., Perisic T., Sato H. (2008). The cystine/cysteine cycle: a redox cycle regulating susceptibility versus resistance to cell death. *Oncogene*.

[3] Hong T., Lei G., Chen X. (2021). PARP inhibition promotes ferroptosis via repressing SLC7A11 and synergizes with ferroptosis inducers in BRCA-proficient ovarian cancer. *Redox Biology*.

[4] Wang L., Liu Y., Du T. (2020). ATF3 promotes erastin-induced ferroptosis by suppressing system Xc^−^. *Cell Death and Differentiation*.

[5] Gao R., Kalathur R. K., Coto‐Llerena M. (2021). YAP/TAZ and ATF4 drive resistance to sorafenib in hepatocellular carcinoma by preventing ferroptosis. *EMBO Molecular Medicine*.

[6] Wang X., Chen Y., Wang X. (2021). Stem cell factor SOX2 confers ferroptosis resistance in lung cancer via upregulation of SLC7A11. *Cancer Research*.

[7] Jiang L., Kon N., Li T. (2015). Ferroptosis as a p53-mediated activity during tumour suppression. *Nature*.

[8] Wang Y., Yang L., Zhang X. (2019). Epigenetic regulation of ferroptosis by H2B monoubiquitination and p53. *EMBO Reports*.

[9] Zhang Y., Shi J., Liu X. (2018). BAP1 links metabolic regulation of ferroptosis to tumour suppression. *Nature Cell Biology*.

[10] Zhang W., Sun Y., Bai L. (2021). RBMS1 regulates lung cancer ferroptosis through translational control of SLC7A11. *The Journal of Clinical Investigation*.

[11] Liu T., Jiang L., Tavana O., Gu W. (2019). The deubiquitylase OTUB1 mediates ferroptosis via stabilization of SLC7A11. *Cancer Research*.

[12] Ma L., Chen T., Zhang X. (2021). The m^6^A reader YTHDC2 inhibits lung adenocarcinoma tumorigenesis by suppressing SLC7A11-dependent antioxidant function. *Redox Biology*.

[13] Ma L., Zhang X., Yu K. (2021). Targeting SLC3A2 subunit of system X_C_^−^ is essential for m^6^A reader YTHDC2 to be an endogenous ferroptosis inducer in lung adenocarcinoma. *Free Radical Biology & Medicine*.

[14] Song X., Zhu S., Chen P. (2018). AMPK-mediated BECN1 phosphorylation promotes ferroptosis by directly blocking system X_c_^−^ activity. *Current Biology*.

[15] Lang X., Green M. D., Wang W. (2019). Radiotherapy and immunotherapy promote tumoral lipid oxidation and ferroptosis via synergistic repression of SLC7A11. *Cancer Discovery*.

[16] Yang J., Zhou Y., Xie S. (2021). Metformin induces ferroptosis by inhibiting UFMylation of SLC7A11 in breast cancer. *Journal of Experimental & Clinical Cancer Research*.

[17] Ding C., Ding X., Zheng J. (2020). miR-182-5p and miR-378a-3p regulate ferroptosis in I/R-induced renal injury. *Cell Death & Disease*.

[18] Wu Y., Sun X., Song B., Qiu X., Zhao J. (2017). miR-375/SLC7A11 axis regulates oral squamous cell carcinoma proliferation and invasion. *Cancer Medicine*.

[19] Drayton R. M., Dudziec E., Peter S. (2014). Reduced expression of miRNA-27a modulates cisplatin resistance in bladder cancer by targeting the cystine/glutamate exchanger SLC7A11. *Clinical Cancer Research*.

[20] Liu X. X., Li X. J., Zhang B. (2011). MicroRNA-26b is underexpressed in human breast cancer and induces cell apoptosis by targeting SLC7A11. *FEBS Letters*.

[21] Yuan J., Liu Z., Song R. (2017). Antisense lncRNA As-SLC7A11 suppresses epithelial ovarian cancer progression mainly by targeting SLC7A11. *Pharmazie*.

[22] Fang X., Cai Z., Wang H. (2020). Loss of cardiac ferritin H facilitates cardiomyopathy via Slc7a11-mediated ferroptosis. *Circulation Research*.

[23] Bersuker K., Hendricks J. M., Li Z. (2019). The CoQ oxidoreductase FSP1 acts parallel to GPX4 to inhibit ferroptosis. *Nature*.

[24] Doll S., Freitas F. P., Shah R. (2019). FSP1 is a glutathione-independent ferroptosis suppressor. *Nature*.

[25] Mao C., Liu X., Zhang Y. (2021). DHODH-mediated ferroptosis defence is a targetable vulnerability in cancer. *Nature*.

[26] Kraft V. A., Bezjian C. T., Pfeiffer S. (2020). GTP cyclohydrolase 1/tetrahydrobiopterin counteract ferroptosis through lipid remodeling. *ACS Central Science*.

[27] Shimada K., Skouta R., Kaplan A. (2016). Global survey of cell death mechanisms reveals metabolic regulation of ferroptosis. *Nature Chemical Biology*.

[28] Abrams R. P., Carroll W. L., Woerpel K. A. (2016). Five-membered ring peroxide selectively initiates ferroptosis in cancer cells. *ACS Chemical Biology*.

[29] Shintoku R., Takigawa Y., Yamada K. (2017). Lipoxygenase-mediated generation of lipid peroxides enhances ferroptosis induced by erastin and RSL3. *Cancer Science*.

[30] Yang W. S., Kim K. J., Gaschler M. M., Patel M., Shchepinov M. S., Stockwell B. R. (2016). Peroxidation of polyunsaturated fatty acids by lipoxygenases drives ferroptosis. *Proceedings of the National Academy of Sciences of the United States of America*.

[31] Doguer C., Ha J. H., Collins J. F. (2018). Intersection of iron and copper metabolism in the mammalian intestine and liver. *Comprehensive Physiology*.

[32] Guo H., Ouyang Y., Yin H. (2022). Induction of autophagy via the ROS-dependent AMPK-mTOR pathway protects copper-induced spermatogenesis disorder. *Redox Biology*.

[33] Gao W., Huang Z., Duan J., Nice E. C., Lin J., Huang C. (2021). Elesclomol induces copper-dependent ferroptosis in colorectal cancer cells via degradation of ATP7A. *Molecular Oncology*.

[34] Jhelum P., Santos-Nogueira E., Teo W. (2020). Ferroptosis mediates cuprizone-induced loss of oligodendrocytes and demyelination. *The Journal of Neuroscience*.

[35] Yang M., Wu X., Hu J. (2022). COMMD10 inhibits HIF1*α*/CP loop to enhance ferroptosis and radiosensitivity by disrupting Cu-Fe balance in hepatocellular carcinoma. *Journal of Hepatology*.

[36] Li Y., Chen F., Chen J. (2020). Disulfiram/copper induces antitumor activity against both nasopharyngeal cancer cells and cancer-associated fibroblasts through ROS/MAPK and ferroptosis pathways. *Cancers*.

[37] Ren X., Li Y., Zhou Y. (2021). Overcoming the compensatory elevation of NRF2 renders hepatocellular carcinoma cells more vulnerable to disulfiram/copper-induced ferroptosis. *Redox Biology*.

[38] Zilka O., Poon J. F., Pratt D. A. (2021). Radical-trapping antioxidant activity of copper and nickel bis(thiosemicarbazone) complexes underlies their potency as inhibitors of ferroptotic cell death. *Journal of the American Chemical Society*.

[39] Tian H., Zhao S., Nice E. C. (2022). A cascaded copper-based nanocatalyst by modulating glutathione and cyclooxygenase-2 for hepatocellular carcinoma therapy. *Journal of Colloid and Interface Science*.

[40] Gou Y., Chen M., Li S. (2021). Dithiocarbazate-copper complexes for bioimaging and treatment of pancreatic cancer. *Journal of Medicinal Chemistry*.

[41] Riegman M., Sagie L., Galed C. (2020). Ferroptosis occurs through an osmotic mechanism and propagates independently of cell rupture. *Nature Cell Biology*.

[42] Takahashi N., Cho P., Selfors L. M. (2020). 3D culture models with CRISPR screens reveal hyperactive NRF2 as a prerequisite for spheroid formation via regulation of proliferation and ferroptosis. *Molecular Cell*.

[43] Yang W. H., Ding C. C., Sun T. (2019). The hippo pathway effector TAZ regulates ferroptosis in renal cell carcinoma. *Cell Reports*.

[44] Hebebrand J., Holm J. C., Woodward E. (2017). A proposal of the European Association for the Study of obesity to improve the ICD-11 diagnostic criteria for obesity based on the three dimensions etiology, degree of adiposity and health risk. *Obesity Facts*.

[45] Frühbeck G., Busetto L., Dicker D. (2019). The ABCD of obesity: an EASO position statement on a diagnostic term with clinical and scientific implications. *Obesity Facts*.

[46] Wang N., Ma H., Li J. (2021). HSF1 functions as a key defender against palmitic acid-induced ferroptosis in cardiomyocytes. *Journal of Molecular and Cellular Cardiology*.

[47] Bai T., Li M., Liu Y., Qiao Z., Wang Z. (2020). Inhibition of ferroptosis alleviates atherosclerosis through attenuating lipid peroxidation and endothelial dysfunction in mouse aortic endothelial cell. *Free Radical Biology & Medicine*.

[48] Li Q., Liu C., Deng L. (2021). Novel function of fluvastatin in attenuating oxidized low-density lipoprotein-induced endothelial cell ferroptosis in a glutathione peroxidase4- and cystine-glutamate antiporter-dependent manner. *Experimental and Therapeutic Medicine*.

[49] Su G., Yang W., Wang S., Geng C., Guan X. (2021). SIRT1-autophagy axis inhibits excess iron-induced ferroptosis of foam cells and subsequently increases IL-1*Β* and IL-18. *Biochemical and Biophysical Research Communications*.

[50] Xin H., Wang M., Tang W. (2016). Hydrogen sulfide attenuates inflammatory hepcidin by reducing IL-6 secretion and promoting SIRT1-mediated STAT3 deacetylation. *Antioxidants & Redox Signaling*.

[51] Zhou Z., Ye T. J., DeCaro E. (2020). Intestinal SIRT1 deficiency protects mice from ethanol-induced liver injury by mitigating ferroptosis. *The American Journal of Pathology*.

[52] Gao J., Li Y., Song R. (2021). SIRT2 inhibition exacerbates p53-mediated ferroptosis in mice following experimental traumatic brain injury. *Neuroreport*.

[53] Jin Y., Gu W., Chen W. (2021). Sirt3 is critical for p53-mediated ferroptosis upon ROS-induced stress. *Journal of Molecular Cell Biology*.

[54] Liu L., Li Y., Cao D. (2021). SIRT3 inhibits gallbladder cancer by induction of AKT-dependent ferroptosis and blockade of epithelial-mesenchymal transition. *Cancer Letters*.

[55] Han D., Jiang L., Gu X. (2020). SIRT3 deficiency is resistant to autophagy-dependent ferroptosis by inhibiting the AMPK/mTOR pathway and promoting GPX4 levels. *Journal of Cellular Physiology*.

[56] Cai S., Fu S., Zhang W., Yuan X., Cheng Y., Fang J. (2021). SIRT6 silencing overcomes resistance to sorafenib by promoting ferroptosis in gastric cancer. *Biochemical and Biophysical Research Communications*.

[57] Qi J., Kim J. W., Zhou Z., Lim C. W., Kim B. (2020). Ferroptosis affects the progression of nonalcoholic steatohepatitis via the modulation of lipid peroxidation-mediated cell death in mice. *The American Journal of Pathology*.

[58] Tsurusaki S., Tsuchiya Y., Koumura T. (2019). Hepatic ferroptosis plays an important role as the trigger for initiating inflammation in nonalcoholic steatohepatitis. *Cell Death & Disease*.

[59] Li X., Wang T. X., Huang X. (2020). Targeting ferroptosis alleviates methionine-choline deficient (MCD)-diet induced NASH by suppressing liver lipotoxicity. *Liver International*.

[60] Liu B., Yi W., Mao X., Yang L., Rao C. (2021). Enoyl coenzyme A hydratase 1 alleviates nonalcoholic steatohepatitis in mice by suppressing hepatic ferroptosis. *American Journal of Physiology. Endocrinology and Metabolism*.

[61] Yang Y., Chen J., Gao Q., Shan X., Wang J., Lv Z. (2020). Study on the attenuated effect of ginkgolide B on ferroptosis in high fat diet induced nonalcoholic fatty liver disease. *Toxicology*.

[62] Katunga L. A., Gudimella P., Efird J. T. (2015). Obesity in a model of _gpx4_ haploinsufficiency uncovers a causal role for lipid-derived aldehydes in human metabolic disease and cardiomyopathy. *Molecular Metabolism*.

[63] Li W., Li W., Leng Y., Xiong Y., Xia Z. (2020). Ferroptosis is involved in diabetes myocardial ischemia/reperfusion injury through endoplasmic reticulum stress. *DNA and Cell Biology*.

[64] Zhang Q., Hu Y., Hu J. E. (2021). Sp1-mediated upregulation of Prdx6 expression prevents podocyte injury in diabetic nephropathy _via_ mitigation of oxidative stress and ferroptosis. *Life Sciences*.

[65] Li D., Jiang C., Mei G. (2020). Quercetin alleviates ferroptosis of pancreatic *β* cells in type 2 diabetes. *Nutrients*.

